# “It’s the difference between life and death”: The views of professional medical interpreters on their role in the delivery of safe care to patients with limited English proficiency

**DOI:** 10.1371/journal.pone.0185659

**Published:** 2017-10-05

**Authors:** Margaret Siyu Wu, Shail Rawal

**Affiliations:** 1 Faculty of Medicine, University of Toronto, Toronto, Ontario, Canada; 2 Division of General Internal Medicine, University Health Network, Toronto, Ontario, Canada; 3 Department of Medicine, University of Toronto, Toronto, Ontario, Canada; Royal Children's Hospital, AUSTRALIA

## Abstract

**Background:**

Patients with limited English proficiency (LEP) experience poorer quality care and more adverse events in hospital. Consequently, there is interest in understanding the role of professional medical interpreters in efforts to improve patient safety.

**Objective:**

To describe the views of professional medical interpreters on their role in the delivery of safe patient care.

**Design:**

Qualitative analysis of in-depth semi-structured interviews.

**Participants:**

15 professional medical interpreters affiliated with the Healthcare Interpretation Network in Toronto, Canada.

**Approach:**

Participants’ views on their role in patient safety were analyzed and organized into themes.

**Key results:**

Professional medical interpreters described being uniquely situated to identify and prevent adverse events involving patients with LEP by: 1) facilitating communication and enhancing patients’ comprehension, 2) giving voice to patients, and 3) speaking up about safety concerns. Participants described a tension between ‘speaking up’ and interpreters’ ethical imperative to remain impartial. Interpreters also highlighted several challenges, including 4) medical hierarchy and healthcare providers’ limited knowledge of the role of interpreters. These challenges introduced safety issues if providers asked interpreters to act outside of their scope of practice.

**Conclusions:**

Our study found that professional medical interpreters view their work as integral to the delivery of safe care to patients with LEP. In order to effectively engage in patient safety efforts together, interpreters and providers require a mutual understanding of their roles. Team hierarchy and limited provider knowledge of the role of interpreters can introduce safety concerns. In addition, interpreters describe a tension between “speaking up” about patient safety and the need for interpreters to remain impartial when facilitating communication. Healthcare institutions, providers, and interpreters must engage in discussion on how to best to “speak up” and integrate interpreters into safety efforts. Importantly, the benefits of partnering with interpreters can only be realized when providers consistently use their services.

## Introduction

Linguistic diversity is a demographic reality in many English-speaking countries. In the United States, more than 20 percent of the population speaks a language other than English at home and 25 million Americans have limited English proficiency (LEP) [1]. There is growing evidence that individuals with LEP experience barriers to safe and high quality care when interacting with the healthcare system. They have longer lengths of stay for some medical and surgical conditions, and are at increased risk of readmission when compared to their English-proficient counterparts [2–4]. Patients with LEP also undergo more unnecessary investigations, report poorer comprehension of discharge instructions, and describe lower satisfaction with the care they receive [5–8].

Importantly, patients with LEP are more likely to experience serious adverse events causing physical harm in hospital when compared to English-proficient patients [9,10]. The most common root cause of serious adverse events reported to the Joint Commission’s Sentinel Event Database is communication errors [11]. Such errors occur at a higher rate in patients with LEP, as language barriers prevent effective communication between patients and healthcare providers [9,12,13]. As a result, patients with LEP may experience a range of adverse events including diagnostic error, medication error, and poor quality informed consent [12,14].

A recent report identified three main causes of medical error in the care of patients with LEP: patients’ cultural beliefs and traditions, healthcare providers’ reliance on their own second language skills, and the use of “ad-hoc interpreters” such as family members or hospital staff for communication [11]. In Canada, the Ontario Council on Community Interpreting (OCCI) defines a professional interpreter as a, “fluently bilingual individual with appropriate training and experience who is able to interpret with consistency and accuracy and who adheres to the Standards of Practice and Ethical Principles [15]. When compared to ad-hoc interpreters, professional medical interpreters commit fewer clinically-significant interpretation errors [16]. In addition, the use of professional medical interpreters improves a number of important health outcomes for patients with LEP including patient satisfaction, length of stay, comprehension of discharge instructions, and hospital readmissions [16–18].

Given the central role of communication errors in adverse events involving patients with LEP, there is growing recognition that interpreters play a significant role in reducing medical error and associated medicolegal risk [7,19]. The Agency for Healthcare Research and Quality (AHRQ) and other bodies have advocated that medical interpreters move beyond solely interpreting clinical interactions to actively voicing concerns about patient safety [7]. However, an expanded role for interpreters may conflict with their ethical requirement to remain impartial when facilitating communication, and it is unclear whether interpreters view patient safety as part of their work. As such, we sought to explore the views of professional medical interpreters on their role in patient safety through this qualitative study.

## Methods

### Study design

This study involved in-depth semi-structured qualitative interviews with professional medical interpreters.

### Setting

All interviews took place in Toronto, Ontario. Toronto is the most linguistically diverse city in Canada. Forty-seven percent of residents have a mother tongue other than English or French (the two official languages) and 28 percent of residents speak a language other than English at home at home [20]. Healthcare providers in most community and academic hospitals in the city have access to face-to-face and telephone interpretation services. Interviews were conducted in private rooms where only the interviewers and the participants were present.

### Sampling

Participants were selected through purposeful sampling of professional medical interpreters affiliated with the Healthcare Interpretation Network (HIN), a not-for-profit organization that promotes the standardization and professionalization of interpretation in Ontario. Members include contract interpreters and permanent staff interpreters employed by healthcare organizations. Members provide interpretation services in more than 170 languages to hospitals and community health centres, and many also work outside of the healthcare setting in the insurance, legal, and immigration sectors. HIN has since merged with the OCCI’s Healthcare Division. Participants were recruited by an email describing the study. There were no exclusion criteria.

### Data collection

The authors conducted in-depth, semi-structured interviews with the participants from March 2016 to August 2016. When an interpreter expressed interest in participating in our study, a mutually convenient time was established for the interview. Interviews were conducted in person and were approximately one hour in duration. The lead author (MW) was a female MD candidate with the University of Toronto at the time of the interviews. There was no relationship between MW and the participants. Written informed consent was obtained from the participants and the lead author (MW) conducted the majority of interviews.

An interview guide was developed prior to beginning the study to inform questioning (S1 File). Participants were asked several open-ended questions and the responses directed further questioning. This method provided sufficient guidance to the interviewee and allowed the interviewer to explore a wide range of factors. The initial interview guide was modified during the study to explore new themes as they emerge in analysis. Interviews were digitally recorded and transcribed verbatim by the authors. In total, fifteen interviews were conducted, at which point the authors determined that no new concepts had arisen in consecutive interviews and theoretical saturation had been reached [21,22].

### Data analysis

Data analysis occurred in parallel with the interviews. Grounded theory informed the analysis and the NVivo 10 software was used to assist with data management. Transcripts were reviewed several times and text related to a concept were identified and coded. Similar codes were then grouped under overarching themes.

The two authors engaged in the process of coding independently to ensure that results could be compared and triangulated. Analysis was an iterative process involving meetings of the authors to discuss codes and integrate analyses. The themes were then organized to create a descriptive framework. Finally, participants were asked to review the study results and to provide any comments or corrections.

### Ethics

This study was approved by the University Health Network Research Ethics Board.

## Results

A total of fifteen professional medical interpreters were interviewed. Participant characteristics are summarized in Table 1. Participants’ views on their role in patient safety were organized into four themes that emerged from the data: 1) facilitating communication and enhancing patients’ comprehension, 2) giving voice to patients, 3) speaking up about safety concerns, and 4) navigating challenges in fulfilling their role.

**Table 1:** Participant characteristics (n = 15).

Characteristic	Categories	Result (%)
**Sex**		
	Male	4 (27)
	Female	11 (73)
Language interpreted*		
	Spanish	3 (20)
	Cantonese	2 (13)
	Mandarin	2 (13)
	Italian	2 (13)
	Vietnamese	2 (13)
	Other	8 (53)
**Number of years working as interpreter**		
	<5 years	2 (13)
	5–10 years	5 (33)
	10–20 years	5 (33)
	>20 years	3 (20)
**Current employment status**		
	Staff interpreter	7 (47)
	Contract interpreter	8 (53)

*There were more than 15 total languages interpreted (>100%) as some interpreters interpreted multiple languages.

### 1. Facilitating communication and enhancing comprehension

Interpreters described their primary role as enabling communication between individuals who would otherwise be unable to communicate effectively: patients with LEP and healthcare providers. In particular, participants highlighted their role in facilitating high-risk communication such as informed consent, medication reconciliation, and discharge counseling.

Participants viewed interpretation as integral to obtaining an accurate clinical history. Such communication was seen to inform the diagnostic process and development of a care plan. In the absence of an interpreter, patients with LEP were felt to be at risk for diagnostic error, low-value investigations, and treatment delays. With the aid of an interpreter healthcare providers:

Will be able to assess the problem quickly… and after diagnosing, will be able to order the tests that are necessary because there is no guessing game. And then afterwards, discharge the patient and give the right medications and send them home with the right instructions so they are not coming back for another visit… Initially it does take more time, but then you actually save time and money afterwards. Not only time and money, but you could be saving a life at the end

(14)

Interpreters also described improving a patient’s understanding of their health and healthcare as integral to ensuring patient safety. One interpreter observed:

The impact of having interpreters? Oh, I think it's the difference sometimes between life and death! You know, you have someone going in an appointment without an interpreter and English is their second language… they are just piecing stuff together. They walk away without knowing what the next steps are, they walk away without knowing what food to eat, how to take their medications properly… They could walk out the door and do serious harm to themselves because they don't have the right information

(6)

Several participants described the importance of involving professional medical interpreters rather than relying on the skills of ad-hoc interpreters. One participant highlighted the risk of communication errors when using ad-hoc interpreters, noting that:

The outcomes of those situations could be devastating. That someone's not qualified and doing the interpretation can drastically change the outcome of the understanding, of the patient's understanding, of the situation. Or it could alter their decision-making for treatment. Potentially, it will cost their life, right? Or lead to a medical error or a liability lawsuit, right?

(8)

### 2. Giving voice to patients

Interpreters highlighted their role in giving voice to patients as a key contribution to patient safety. If LEP patients have a voice in clinical encounters with their healthcare providers, then they themselves may raise safety concerns. One participant noted that,

I think that we are invaluable to the experience of patients here. It can make or break a person's experience, if they are able to express themselves, say the things that they need, and get an understanding of their own care, they can take back their independence. Isn't that the first order of business? Instead of the last, as an afterthought, you know?

(6)

Some participants viewed their work as important to creating a climate that allowed patients with LEP to feel comfortable speaking up about safety concerns.

It's intimidating to speak up when you have a doctor. So, if the doctor says something, even if you're not convinced and even if they ask… “if you want to ask anything?” It's always intimidating to say something, because you think he's going to get upset or the care is not going to be the same level… I try to get the best level of communication as possible…So sometimes the patient might have more difficulty hearing, but doesn't want to wear a hearing aid, doesn't want to wear headphones, but he loves to read… so in Italian, writing may be easier to interpret

(5)

Another participant described the role of interpreters as balancing the asymmetry in power between health professionals and LEP patients. An empowered patient was viewed as an active participant with a voice in their care.

If you don’t have a voice, you are powerless. And if you cannot communicate what is going on, you are… you cannot do anything. They will just decide for you. It’s horrible. It’s horrible. If you have a voice, and it’s an impartial voice, and it says only what you say, exactly what you say, exactly how you say it… then, they will be able to choose or to access, and so I think the balance is… improved. If not, it is like the doctor is powerful and the patient is powerless because they don’t have a voice

(15)

### 3. Speaking up about safety concerns

As impartial actors, interpreters are uniquely situated to identify safety concerns in interactions between patients and healthcare providers. In their role as both an insider and outsider to the healthcare team, they may identify safety issues that are not noted by others. One participant observed,

I engage in patient safety as much as any other profession in the hospital, in the medical field. So, with the language gap that we are trying to bridge in a way we are providing extra safety, because we are able to communicate whatever concern… to the provider, mainly with the language that the patient speaks, right? But in the broader spectrum, we are engaged in making sure that patient safety is always the first thing, even if it's not necessarily related to interpreters, like we might catch other situations…on the sidelines, that we can catch working as interpreters that they may be missing, you know?

(5)

Some participants viewed speaking up about patient safety concerns as part of their role. One noted that,

If I feel the doctor, the service provider, is going to make a mistake, and I know some stuff from working before with the patient, then I have to… it's my role to mention it right? So… let's say my client, she's going to surgery for her finger, and I can see the nurse talking and they're talking with another nurse, and they are getting the client ready for a heart surgery, then I have to say something, right?

(2)

Some felt that concerns about patient safety should be raised in a manner that was deferential to the healthcare provider. A participant noted,

You have to deal with this [potential error] in a more diplomatic way. I will pretend that maybe I misunderstood. And then I will raise my hand and ask, “do you mean…two times a day, three tablets? Or three times a day?” Or whatever. You make it sound like that you want to clarify and kind of remind them [healthcare providers], hint at them that “was this the right message that you wanted me to interpret?” So, doing that you kind of deescalate the situation, and you make sure that both ends receive the same information. That's why I say that we are bridging, in a way. That if we saw something that's out of order, we have to interfere, and that's something we can do to prevent medical errors that are going to happen

(8)

Interpreters also reported struggling with decisions to “speak up” about patient safety concerns. One interpreter felt that the decision to raise a safety concern was, *“a judgment call with every single situation”*. Speaking up was viewed by some as transgressing the boundaries of their role and in conflict with a code of conduct that requires interpreters to remain impartial in clinical encounters and to interpret only what is said.

I take a chance sometimes to be borderline, ‘cause we have a pretty strict protocol and procedure. Like I said, we're supposed to do [interpret] and that's it. But if something comes to my attention, it's hard for me to keep it. If I, you know, get the sense it might be relevant… I try to bring up the issue

(5)

Interpreters also described the relationship between raising patient safety concerns and medical hierarchy. After speaking up about a patient safety issue, one interpreter feared that, *“maybe next time when you go through that clinic*, *you are not welcome*. *You're a whistleblower*, *and you brought some trouble to them (7)*.*”*

### 4. Navigating challenges in fulfilling their role

While interpreters felt that they played a significant role in facilitating safe and high quality patient care, they also highlighted several important barriers. Interpreters often felt like they were not part of the healthcare team and this limited their ability to effectively fill their role.

As an interpreter, you are in the periphery… you are not part of the core team that's making the decision. But it's important that we are still part of the team so that we know the content of the information that's going to be discussed so we can represent the information accurately and effectively

(13)

Participants also reported that established hierarchy amongst healthcare providers made it difficult to raise patient safety concerns.

Well we are a pain in the neck for [healthcare providers] … they are sometimes very thankful, many times. But it's like a class thing…sometimes it's hard. I think it's a profession that is not totally valued

(8)

Others described potential patient safety concerns that could arise from healthcare providers’ poor understanding of the role of interpreters. Some interpreters reported being asked to complete consent forms independently with patients and others noted that healthcare providers would sometimes ask for their opinion on a patient’s diagnosis. One described that,

The first thing is always, "what is your opinion, what do you think?" Unfortunately, [diagnosing] is not what I should do. it's really unprofessional of me so I would never do that…Even if I know, it doesn't matter, you have to express what you want to say and it's my job to interpret everything…Some people are not familiar with interpretation and it might be human, you know, to say “what do you think”, but it's not our role at all, it would never be

(5)

## Discussion

Patients with limited English proficiency experience a range of safety issues when hospitalized. There is growing evidence that professional medical interpreters improve the quality of care received by patients with LEP and there is interest in engaging interpreters in patient safety efforts.

Our study found that interpreters felt they play a significant role in assisting healthcare providers in delivering safe and high quality care to patients with LEP. Interestingly, this role had several dimensions that extended beyond simply bridging the communication gap between patients and healthcare providers. Interpreters viewed their work as integral to ensuring that patients understood the care they were about to receive and could engage in treatment recommendations and decisions. In particular, they highlighted their impact on reducing hazards inherent to high-risk tasks such as diagnosis, medication counseling, care transitions, and the informed consent process [7].

Importantly, some interpreters described “speaking up” when they saw significant patient safety issues as a component of their role in promoting patient safety. They fulfilled this function in a number of ways, ranging from simply altering the tone in which they interpreted, for example by framing a statement as question to prompt clarification, to explicitly raising a safety concern with a healthcare provider. Speaking up required that interpreters navigate the boundaries of their role as they are typically trained to act as a *conduit*, a model that requires that the interpreter aim to become "invisible" in the interaction between patient and healthcare provider [23].

However, the complexity of a clinical encounter can make it difficult to operationalize an impartial position, and interpreters may act outside of this role to resolve conflicts and advocate for a patient [24,25]. In our study, some participants expressed a sense of conflict about “speaking up” and viewed it as acting outside the boundaries of their role. Some also expressed the view that the responsibility for ensuring the integrity of the communication and the safety of care delivered rests with the healthcare provider, and interpreters ought not to ‘speak up’.

In order to function as impartial actors in the interaction between the patient and healthcare provider, interpreters are necessarily situated outside of the healthcare team. And yet, they work within hierarchical team environments where not all healthcare providers understand or value the role of professional medical interpreters. It is not surprising then that several interpreters’ highlighted challenges related to hierarchy as barriers to raising patient safety concerns. Our findings also suggest that providers who lack knowledge about the role of interpreters may compromise patient safety by asking interpreters to engage in processes outside of their scope of practice, such as providing an opinion on a diagnosis or independently obtaining informed consent from patients. Healthcare providers may also assume that care is safer simply because an interpreter is present, rather than working to ensure that the care delivered to patients with LEP is safe.

Consistent with the literature, interpreters also highlighted the importance of being engaged in the care of patients with LEP and described the risks of overreliance on ad-hoc interpreters or no interpreters at all. In particular, they stressed the significance of giving voice to LEP patients so that patients themselves might participate in their healthcare and help to ensure that the care delivered is safe. Our results demonstrate that interpreters view patient safety as a key component of their work, but have trouble operationalizing their commitment to safety due to the constraints of their role.

The primary limitation of our work is its generalizability. Our findings represent an analysis of the views of the 15 professional medical interpreters working in Toronto and as such, may not be generalizable to other settings. In addition, participants may have been inclined to highlight the positive aspects of their involvement in the care of patients with LEP.

There are several potential next steps to begin leveraging the unique skillset of professional medical interpreters in patient safety efforts. First, there is a need for healthcare providers to understand the potential benefits of partnering with professional medical interpreters in delivering care to patients with LEP. Second, there is an opportunity for healthcare institutions to provide medical interpreters with training in patient safety theory and orientation to institutional safety efforts. Third, healthcare providers, institutions, and interpreters must engage in conversations about the role of interpreters in “speaking up” about patient safety issues to determine how to balance the need for interpreters to remain impartial and their unique perspective on patient safety events. Formal programs like the *AHRQ TeamSTEPPS Enhancing Safety for Patients with Limited English Proficiency Module* may represent a way forward [26]. Fourth, even in settings with robust interpretation services, interpreters are used in less than one third of encounters with LEP patients [27]. Thus, any initiative to leverage the skillset of professional interpreters in patient safety will require interventions to increase the use interpreters by healthcare providers.

## Conclusion

Our study found that professional medical interpreters view their work as integral to the delivery of safe care to patients with limited English proficiency. In order to effectively engage in patient safety efforts together, interpreters and healthcare providers require a mutual understanding of their roles. Team hierarchy and limited provider knowledge of the role of interpreters can introduce safety concerns. In addition, interpreters describe a tension between “speaking up” about patient safety and the need for interpreters to remain impartial when facilitating communication. Healthcare institutions, providers, and interpreters must engage in discussion on how to best to “speak up” and integrate interpreters into safety efforts. Importantly, the benefits of partnering with interpreters can only be realized when healthcare providers consistently use their services.

## Supporting information

S1 File Initial interview guide. (DOCX)
