# Supplementary material for: “It’s the difference between life and death”: The views of professional medical interpreters on their role in the delivery of safe care to patients with limited English proficiency
Source: PLoS One. 2017 Oct 5;12(10):e0185659. doi: 10.1371/journal.pone.0185659 (PMC5628836; doi:10.1371/journal.pone.0185659)
Supplement: S1 File — (DOCX) [file pone.0185659.s001.docx]

**S1 Appendix**

**Initial Interview guide**

1) Can you tell me about your work as a professional medical interpreter?

2) Are there any issues related to the safety and quality of healthcare that you have observed?

3) How do you think your role relates to the safety of patients?

4) How do you think your role relates to the delivery of high quality healthcare?

5) What are your thoughts on the role of professional medical interpreters and the healthcare team?
